# High-Throughput, Sequence-Based Analysis of the Microbiota of Greek Kefir Grains from Two Geographic Regions

**DOI:** 10.17113/ftb.58.02.20.6581

**Published:** 2020-06

**Authors:** Mary S. Kalamaki, Apostolos S. Angelidis

**Affiliations:** 1 Division of Science & Technology, American College of Thessaloniki, 17 Sevenidi Street, 55510 Thessaloniki, Greece; 2 Department of Hygiene and Technology of Food of Animal Origin, School of Veterinary Medicine, Faculty of Health Sciences, Aristotle University of Thessaloniki, 54124 Thessaloniki, Greece

**Keywords:** kefir, bacterial diversity, species richness, high-throughput sequencing, probiotic drink

## Abstract

**Research background:**

Kefir is a natural probiotic drink traditionally produced by milk fermentation using kefir grains. Kefir grains are composed of a complex population of bacteria and yeasts embedded in a polysaccharide-protein matrix. The geographic origin of kefir grains may largely influence their microbial composition and the associated kefir drink properties. Although the detailed bacterial composition of kefir grains from several geographic regions has been reported, to date, analogous data about the microbiome of Greek kefir are lacking. Hence, the aim of this study is to investigate the structure and the diversity of the bacterial community of Greek kefir grains.

**Experimental approach:**

The bacterial community structure and diversity of two different kefir grains from distant geographic regions in Greece were examined *via* high-throughput sequencing analysis, a culture-independent metagenomic approach, targeting the 16S rRNA V4 variable region, in order to gain a deeper understanding of their bacterial population diversities.

**Results and conclusions:**

Firmicutes (a phylum that includes lactic acid bacteria) was strikingly dominant amongst the identified bacterial phyla, with over 99% of the sequences from both kefir grains classified to this phylum. At the family level, Lactobacillaceae sequences accounted for more than 98% of the operational taxonomic units (OTUs), followed by Ruminococcaceae, Lahnospiraceae, Bacteroidaceae and other bacterial families of lesser abundance. Α relatively small number of bacterial genera dominated, with *Lactobacillus kefiranofaciens* being the most abundant in both kefir grains (95.0% of OTUs in kefir A and 96.3% of OTUs in kefir B). However, a quite variable subdominant population was also present in both grains, including bacterial genera that have been previously associated with the gastrointestinal tract of humans and animals, some of which are believed to possess probiotic properties (*Faecalibacterium* spp., *Bacteroides* spp., *Blautia* spp.). Differences among the bacterial profiles of the two grains were very small indicating a high homogeneity despite the distant geographic origin.

**Novelty and scientific contribution;:**

This is the first study to deeply explore and report on the bacterial diversity and species richness of Greek kefir.

## INTRODUCTION

Kefir is a fermented dairy product with probiotic properties. It is mildly acidic, self-carbonated, with a creamy consistency and a unique flavour attributed to the bacterial and yeast fermentation products. The reported health benefits to the consumer are proposed to be associated with biochemical alterations in milk constituents during milk fermentation, such as the production of bioactive peptides and organic acids, and with the presence of probiotic microorganisms (*1*, *2*). It is traditionally produced by inoculation of milk (primarily bovine milk, although other milk types can be used) with kefir grains, which comprise symbiotic communities of bacteria and yeast that are embedded in a polysaccharide-protein matrix. Kefir grains contain a variety of microbiota including lactic acid bacteria (LAB) and yeast, and occasionally acetic acid bacteria (*3*-*7*). Kefir grains of different origin contain distinct consortia of microorganisms (*3*, *7*) and the microbial composition of kefir of different geographic origins has been investigated using culture-dependent and/or culture-independent methods (*4*, *8*-*10*).

Metagenomics provides a powerful tool for the analysis of microbial communities that does not depend on culturing and has been increasingly used in studies involving food microbial communities (*11*). Hence, in the last decade, metagenomic analyses have been employed for the culture-independent, in-depth description of the microbial communities of kefir grains from Ireland (*7*, *12*), Brazil (*13*), Tibet (*14*, *15*), Turkey (*16*, *17*), USA, Spain, Canada and Germany (*7*), Italy (*7*, *18*), Belgium (*19*), Malaysia (*20*), France and the UK (*7*, *21*).

In Greece, with the exception of one published study focusing on the fungal composition of kefir grains and kefir drinks (*22*), to date there is no literature on the composition of the Greek kefir microbial community. In particular, the bacterial diversity in Greek kefir grains has not been studied. The aim of this study is to explore the bacterial diversity and species richness of two kefir grains originating from distant geographic regions in Greece using a metagenomic approach. This is the first report on using metagenomic analysis to elucidate the microbiological composition of Greek kefir grains.

## MATERIALS AND METHODS

### Kefir grain samples

Kefir grains were obtained from two geographically distant artisanal kefir producers in Greece, located in Athens (kefir A) and Crete (kefir B). The sampling of grains was done aseptically; grains were transported to the laboratory in low-fat (1.5%) ultra-high temperature (UHT) milk under refrigeration (approx. 5 °C). Upon arrival to the laboratory, the grains were propagated in low-fat UHT milk at the 5% inoculation level (*m*/*V*) and incubated at 25 °C for 24 h. At the end of fermentation, the grains were filtered through a sterile sieve and washed with sterile normal saline. This grain propagation procedure was repeated five times and then the kefir grains were used for DNA extraction.

### DNA isolation and high-throughput sequencing

Total DNA isolation was performed using 10 g of kefir grains that were placed in a stomacher filter bag with 90 mL of ¼ strength Ringer’s solution (Lab M Limited, Lancashire, UK). The sample was mixed at maximum speed in a Stomacher 400 Lab blender (Seward Medical, London, UK) for 15 min. The liquid was centrifuged at 17 590×*g* and 4 °C (model 7780; Kubota Corp., Tokyo, Japan) for 7 min. The pellet was resuspended in 20 mM Tris, 2 mM EDTA, 1% Triton X-100 and 30 mg/mL lysozyme (Merck, Darmstadt, Germany) and incubated at 37 °C for 1 h. After the incubation period, 200 μL were removed to a sterile microcentrifuge tube and 20 μL of proteinase K (Thermo Fisher Scientific Inc., Rochester, NY, USA) were added. The solution was incubated overnight at 55 °C. After this step, DNA was extracted using the GeneJET Whole Blood Genomic DNA Purification mini kit (Thermo Fisher Scientific Inc.) according to the manufacturer’s instructions. The concentration and purity of the extracted DNA were determined in a SmartSpec™ Plus spectrophotometer (BioRad Inc., Hercules, CA, USA).

One hundred ng of each DNA sample were used for a polymerase chain reaction (PCR) using the HotStarTaq *Plus* Master Mix Kit (Qiagen, Valencia, CA, USA). Primers 515F (5′-GTGCCAGCMGCCGCGGTAA-3′) and 806R (5′-GGACTACVSGGGTATCTAAT-3′) that target the 16S rRNA V4 variable region were used with a barcode on the forward primer (*23*). PCR conditions were as follows: 94 °C for 3 min, followed by 28 cycles of 94 °C for 30 s, 60 °C for 40 s and 72 °C for 1 min, and a final elongation step at 72 °C for 5 min. Successful amplification was determined on a 2% agarose gel. Samples were purified using calibrated Ampure XP beads (Agencourt Bioscience Corporation, Danvers, MA, USA). Purified PCR products were used to prepare a DNA library by following the Illumina TruSeq DNA library preparation protocol (Illumina Inc., San Diego, CA, USA). Sequencing was performed at the Molecular Research Laboratory, Shallowater, TX, USA on an Illumina MiSeq instrument following the manufacturer’s guidelines.

### Processing of sequencing data

Sequence data were processed using a commercial sequencing facility analysis pipeline (Molecular Research Laboratory) (*23*). In brief, sequences were joined and edited to remove the barcode and primer sequences. Sequences less than 150 bp and sequences with ambiguous base calls were removed. Next, sequences were denoised and clustered at 3% divergence to generate operational taxonomic units (OTUs), followed by the removal of chimeric sequences. Final OTUs were taxonomically classified using BLASTn (*24*) against a curated database derived from GreenGenes (*25*), Ribosomal Database Project (RDPII) (*26*) and the National Center for Biotechnology Information (NCBI) (*27*). All the sample raw reads have been deposited at NCBI and are available under the BioProject ID PRJNA635224.

### Diversity assessment

Diversity indices were calculated at the species level (*28*). The Shannon entropy (*H*') was calculated as follows:


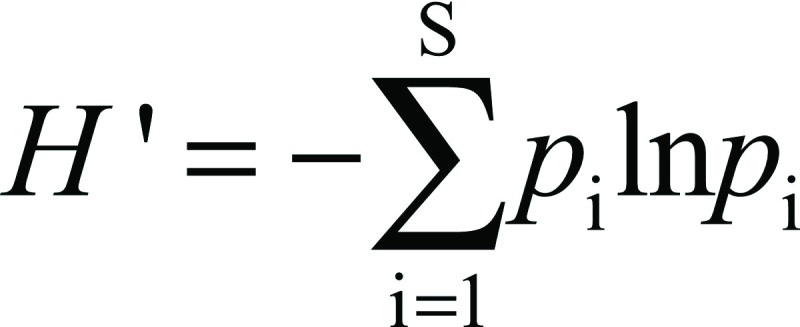


where *p*_i_ is the proportion of species *i* relative to the total number of species, and *S* is the total number of species. Shannon's equitability (*E*_H_) was calculated by dividing *H*' by *H*'_max_ (were *H*'_max_=ln*S*).


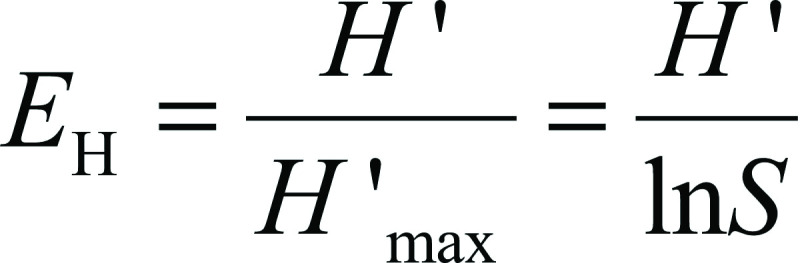


The Simpson (*D*) and Gini-Simpson's (*D*_GS_) diversity indices were calculated using the following equations, respectively:


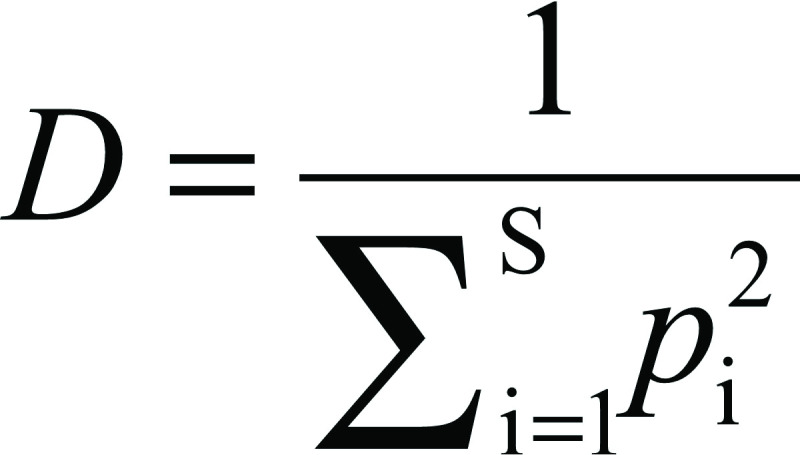


And


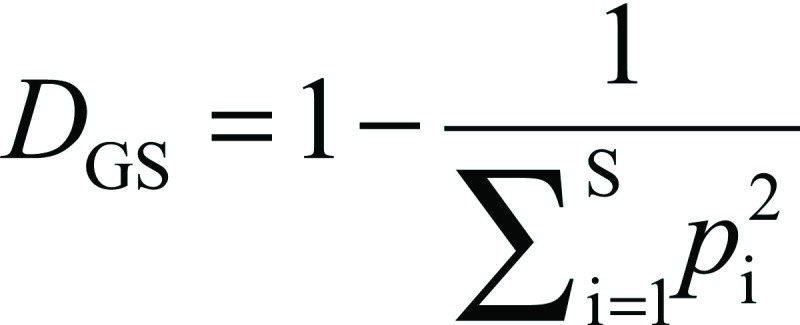


Hill numbers were calculated using the equation:


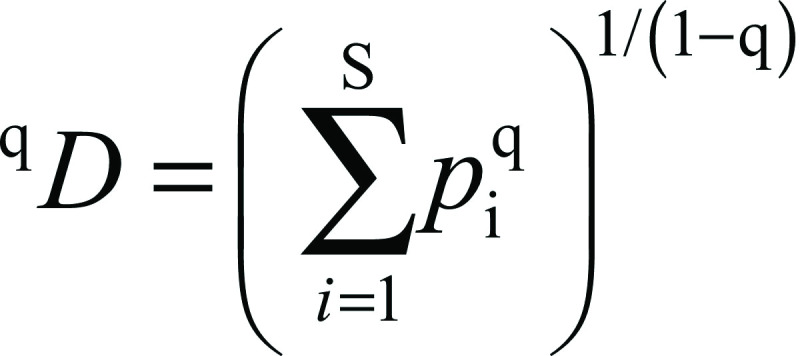


where q is the order of the diversity, a parameter that controls the sensitivity of the measure to the relative abundance of the species (*29*, *30*). For q=0 the Hill number indicates species richness; in the limit as q approaches 1, the Hill number represents the exponential of Shannon's entropy index, whereas for q=2 it signifies the inverse of Simpson's concentration index. Alpha and beta diversity data were generated using QIIME (*31*). A principal coordinate analysis (PCoA) plot based on the unweighted UniFrac distance matrix was generated using EMPeror in QIIME (*32*).

## RESULTS AND DISCUSSION

### Sequencing results

The analysis of the microbiota of the two kefir grains by 16S amplicon sequencing generated a total of 305 072 raw sequences. Of these, 156 370 were from kefir A and 148 702 belonged to kefir B.

### Bacterial diversity of Greek kefir grains

**Fig. 1:**
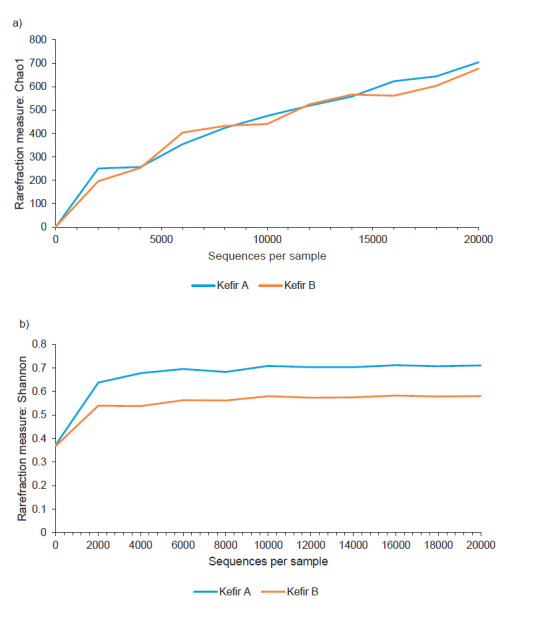
Rarefaction curves of both samples showed a plateau as the number of sequences increased indicating that the kefir community was sufficiently sampled: a) Chao1 is a non-parametric richness estimator, which estimates the number of species present as singletons or doubletons in a sample based on abundances and has units of number of species, b) Shannon index is an entropy measure, which provides the uncertainty in the species diversity of a randomly chosen individual in the community and has units of bits of information. Blue line represents kefir A (Athens) and red line represents kefir B (Crete)

**Fig. 2:**
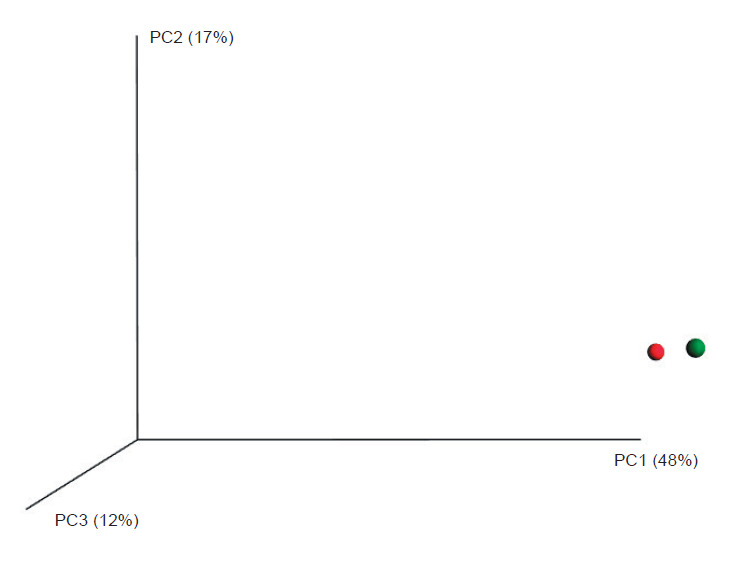
Principal coordinate analysis plot based on unweighted UniFrac distance matrices for kefir A (Athens, red circle) and kefir B (Crete, green circle). UniFrac measures the phylogenetic distance between sets of taxa in a phylogenetic tree as a fraction of branch length. This metric captures the total amount of evolution that is unique to each sample, reflecting adaptation to one environment that could be deleterious to the other. The percentages in the axis labels represent the percentages of variation explained by the principal coordinates

Diversity metrics in microbiome studies is used to infer the structure of a community with respect to species richness and evenness. Rarefaction curves (Fig. 1) of both samples showed a plateau as the number of sequences increased, indicating that the bacterial community of the two grains was adequately sampled. Diversity indices provide information about the composition of a community by considering the relative abundances of different species. Species richness is a measure of the total number of different species in a sample. At the species level, the microbiota of kefir A was composed of 61 distinct species versus 55 in kefir B. Since species richness does not incorporate any information about the relative abundance of a species, values of the Shannon entropy, Shannon's equitability, Simpson dominance and Gini-Simpson index were calculated and are reported in Table 1. A Shannon entropy index of 1 indicates that all species are equally represented in the bacterial community of a sample, whereas a high value of the Simpson’s index indicates low diversity. Based on the value for Shannon entropy (0.24 for kefir A and 0.18 for kefir B) and Simpson dominance (0.92 for kefir A and 0.94 for kefir B), we could describe the equitability, or evenness of individual distributions among species in the Greek kefir grain community, as relatively low. This is in line with other studies reporting on the presence of one or a few dominant species in kefir grain communities from different geographic locations (*7*, *13*, *18*, *33*). Since both indices have limitations, the effective number of species, expressed by Hill numbers, was proposed as more informative metrics to quantify diversity (*29*, *30*). Hill numbers were calculated for the two kefir grain samples and are presented in Table 2. As the order of q increases, low diversity values indicate a high degree of dominance in the community (*29*). As shown, the effective number of species, a metric that is better associated with dominance, was 1.27 for kefir A and 1.20 for kefir B. Based on the calculated diversity indices, the bacterial community in the two Greek kefir grains shows high dominance by a few bacterial species. Beta diversity (a measure of the difference between the entire microbial community in kefirs A and B) provides complementary information on community variation. Beta diversity was assessed by calculating the UniFrac distance metric, which is based on the fraction of branch length within a phylogenetic tree that is shared between two bacterial communities. PCoA of the microbial community of each sample based upon the unweighted (abundance independent) UniFrac distance matrix was performed in order to compare the diversity in the microbial composition between the two samples. Unweighted UniFrac considers only the presence or absence of lineages and provides information on community membership. The PCoA plot illustrated in Fig. 2. shows that the microbiota in the two kefir grains cluster together, indicating that the two microbial communities are evolutionarily similar.

**Table 1:** Alpha diversity indices for kefir A and B

	Kefir A		Kefir B
Richness	61		55
Shannon entropy	0.237		0.181
Shannon's equitability	0.058		0.045
Simpson dominance	0.920		0.944
Gini-Simpson index	0.080		0.057

Kefir A grain originated from Athens and kefir B from Crete

**Table 2:** Hill numbers for the order of q for kefir A and B

	Hill number (^q^*D*)		
Order q	Kefir A		Kefir B
0	61		55
1	1.27		1.20
2	1.09		1.06
3	1.07		1.04
4	1.06		1.04
5	1.04		1.03

Hill numbers (^q^D) measure diversity as the effective number of species. Hill number for q=0 indicates species richness, q=1 represents the exponential of Shannon's entropy index and q=2 the inverse of Simpson's concentration index. Kefir A grain originated from Athens and kefir B from Crete

### Bacterial profile of Greek kefir grains

**Fig. 3:**
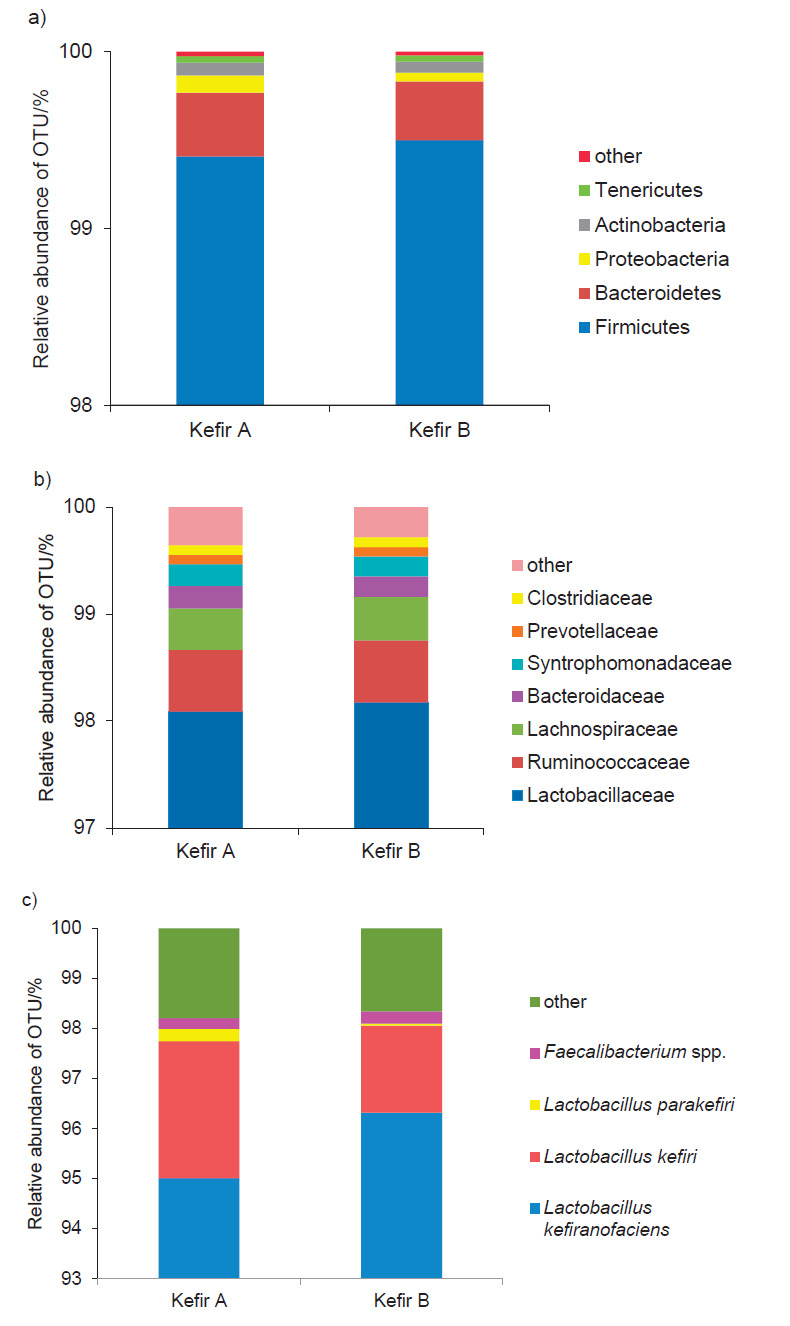
Relative abundance of bacterial OTUs (operational taxonomic units) at the a) phylum, b) family and c) genus/species levels detected by 16S metagenomic analysis of two kefir grains of distant geographic origin in Greece: kefir A (Athens) and kefir B (Crete)

The percentages of bacterial OTUs assigned to the phylum, family, genus and species levels of taxonomy are given in Fig. 3. The bacterial phylum Firmicutes, which includes LAB, dominated the bacterial OTUs, with 99.4 and 99.5% of the sequences of kefir A and kefir B, respectively, classified to this phylum (Fig. 3a). Lactobacillaceae was the dominant bacterial family in both kefir grains (approx. 98%) followed by Ruminococcaceae (0.6%), Lachnospiraceae (0.4%), Bacteroidaceae (0.2%), Syntrophomonadaceae (0.2%) and other families of minor representation (Fig. 3b).

The predominant species identified in both kefir grains was *Lactobacillus kefiranofaciens*, followed by *Lb. kefiri* and *Lb. parakefiri*. The percentages of OTUs assigned to these species were different in the two kefir grains, as shown in Fig. 3c. *Lb. kefiranofaciens* was more prevalent in kefir B (96.3 *vs*. 95.0% in kefir A), whereas *Lb. kefiri* and *Lb. parakefiri* OTUs were identified at higher percentages in kefir A (2.75 and 0.25% in kefir A, *vs*. 1.74 and 0.04% in kefir B). All three species are described as normal and frequent components of kefir drinks and kefir grains (*12*, *18*, *19*). Specifically, *Lb. kefiranofaciens* is classified as a homofermentative LAB (*34*), producing principally d-(–)-lactic acid (without gas) from glucose fermentation, and has been identified as the most abundant bacterial species in kefir grains originating from different parts of the world (*13*, *16*, *18*-*20*, *35*). *Lb. kefiranofaciens* produces kefiran, a biopolymer (polysaccharide) possessing a variety of health benefits (anti-inflammatory, antibacterial, antitumour, antioxidant) and technological functionalities due to its favourable characteristics, such as its rheological behaviour, biocompatibility and emulsifying properties (*36*). *Lb. kefiri* and *Lb. parakefiri* (heterofermentative LAB) are also common kefir isolates around the world (*13*, *19*). *Lb. kefiranofaciens* and *Lb. kefiri* are considered as two key LAB in the mechanism of kefir grain formation (*37*). OTUs of other species of lactobacilli that were recovered in very low or rare frequencies included *Lb. crispatus*, *Lb. aviarius*, *Lb. agilis* and *Lb. salivarius* (Table 3).

**Table 3:** Numbers and percentages of bacterial operational taxonomic units (OTUs) identified in two kefir grains from distant geographic origin in Greece (kefir A grain originating from Athens and kefir B from Crete)

Bacterial genus or species	*N*(bacterial OTU)			*N*(bacterial OTU)/%	
Kefir A	Kefir B		Kefir A	Kefir B
*Lactobacillus kefiranofaciens*	142970	138125		95.005	96.318
*Lactobacillus kefiri*	4133	2494		2.746	1.739
*Lactobacillus parakefiri*	367	59		0.246	0.041
*Faecalibacterium* spp.	321	351		0.215	0.247
*Bacteroides* spp.	191	159		0.128	0.112
*Pseudobutyrivibrio* spp.	119	120		0.080	0.084
*Clostridium* spp.	118	99		0.079	0.070
*Coprococcus* spp.	96	104		0.064	0.073
*Blautia* spp.	85	100		0.057	0.070
*Subdoligranulum* spp.	81	73		0.054	0.051
*Acinetobacter johnsonii*	96	23		0.064	0.016
*Olsenella* spp.	68	54		0.046	0.038
*Lactobacillus crispatus*	46	28		0.031	0.020
*Allobaculum* spp.	37	22		0.025	0.015
*Ruminococcus* spp.	34	39		0.023	0.027
*Bacteroides barnesiae*	32	31		0.021	0.022
*Anaeroplasma* spp.	28	25		0.019	0.018
*Shuttleworthia* spp.	25	26		0.017	0.018
*Phascolarctobacterium* spp.	24	22		0.016	0.015
*Paraprevotella*	21	23		0.014	0.016
*Lactobacillus aviarius*	20	21		0.013	0.015
*Parabacteroides* spp.	18	12		0.012	0.008
*Oscillospira* spp.	15	9		0.010	0.006
*Alistipes* spp.	13	15		0.009	0.011
*Flavonifractor* spp.	13	8		0.009	0.006
*Moryella*	13	15		0.009	0.011
*Bacteroides salanitronis*	12	15		0.008	0.011
*Lactobacillus* spp.	10	7		0.007	0.005
*Anaerotruncus* spp.	9	8		0.006	0.006
*Sutterella*	9	8		0.006	0.006
*Thalassospira* spp.	8	9		0.005	0.006
*Bacteroides plebeius*	6	1		0.004	0.001
*Slackia* spp.	6	7		0.004	0.005
*Clostridium orbiscindens*	5	7		0.003	0.005
*Papillibacter* spp.	4	3		0.003	0.002
*Roseburia*	4	9		0.003	0.006
*Turicibacter* spp.	4	8		0.003	0.006
*Anaerofilum* spp.	3	3		0.002	0.002
*Bacillus* spp.	3	2		0.002	0.001
*Bacteroides coprocola*	3	3		0.002	0.002
*Clostridium lactatifermentans*	3	1		0.002	0.001
*Leuconostoc mesenteroides dextranicum*	3	0		0.002	0.000
*Mucispirillum* spp.	3	4		0.002	0.003
*Pseudomonas fluorescens*	3	1		0.002	0.001
*Bacteroides coprophilus*	2	7		0.001	0.005
*Collinsella*	2	2		0.001	0.001
*Enterococcus cecorum*	2	4		0.001	0.003
*Lactobacillus agilis*	2	0		0.001	0.000
*Lactobacillus salivarius*	2	1		0.001	0.001
*Marvinbryantia*	2	4		0.001	0.003
*Oscillibacter* spp.	2	3		0.001	0.002
*Veillonella magna*	2	1		0.001	0.001
*Aeriscardovia aeriphila*	1	0		0.001	0.000
*Akkermansia*	1	0		0.001	0.000
*Anaerostipes*	1	0		0.001	0.000
*Bacteroides capillosus*	1	0		0.001	0.000
*Barnesiella* spp.	1	0		0.001	0.000
*Dorea*	1	0		0.001	0.000
*Peptococcus* spp.	1	2		0.001	0.001
*Prevotella* spp.	1	0		0.001	0.000
*Pseudoflavonifractor* spp.	1	0		0.001	0.000
*Fusobacterium mortiferum*	0	1		0.000	0.001
*Megamonas* spp.	0	2		0.000	0.001
*Megasphaera*	0	1		0.000	0.001
*Oxalobacter* spp.	0	1		0.000	0.001

Interestingly, OTUs from a variety of microorganisms which have been previously associated with the human gut microbiome or dairy animals’ rumen were noted at generally small but variable frequencies in our analyses. Hence, a noticeable percentage of OTUs in both kefir grains (approx. 0.22 and 0.25% in kefir A and B, respectively) corresponded to *Faecalibacterium* spp. To date, there is only one recognized species within this genus, *F. prausnitzii*, a butyrate-producing bacterium of the colon, which is considered as beneficial to humans and animals and is found in the mammalian and avian gut, but also occasionally isolated from bovine milk (*38*). To our knowledge, there is only one published study reporting on the presence of *Faecalibacterium* spp. in kefir grains (*7*) at very low frequencies (<1%) in the overall population.

*Bacteroides* spp. ranked third in terms of relative abundance, with approx. 0.13% in kefir A and 0.11% in kefir B. OTUs of six species within the genus *Bacteroides* were noted (albeit in minor to rare frequencies) in the kefir grains analyzed: *B. barnesiae*, *B. coprocola*, *B. coprophilus*, *B. plebeius*, *B. salanitronis* (detected in both kefir grains) and *B. capillosus* (in grain A only). Previously published studies on kefir have revealed sequences belonging to the same family (Bacteroidaceae) level of taxonomy and only one study (*20*) reported sequences to the species level (*B. chinchillae*, *B. stercorirosoris* and *B. vulgatus*). To our knowledge, these six bacterial species have never been previously associated with kefir. Bacteria of the genus *Bacteroides* are strictly anaerobic and reside in the gastrointestinal tract of humans and animals. Owing to their beneficial modulatory mechanisms (interactions with the host), members of *Bacteroides* spp. have received attention as promising candidates for next-generation probiotics (*39*).

A similarly minor frequency of OTUs (approx. 0.08%) across both grains were identified as *Pseudobutyrivibrio* spp., which are members of the Lachnospiraceae family. These organisms are Gram-negative anaerobic rods, which ferment a variety of carbohydrates, with major fermentation end-products being formate, butyrate and lactate. *Pseudobutyrivibrio* spp. are considered commensal bacteria in the rumen of dairy ruminants (*40*).

Similar and very low percentages of *Clostridium* spp. OTUs (0.07-0.08%) were noted in the two grains. At the species level, only rare sequences of *C. orbiscindens* (0.003 and 0.005%) and *C. lactatifermentans* (0.002 and 0.001%) were noted. *C. orbiscindens* is an abundant member of the human gut microbiome (*41*), whereas *C. lactatifermentans* is a member of the clostridial 16S rRNA cluster XIVb, originally described as a chicken caecum isolate (*42*) and was recently proposed to be re-classified in the novel genus *Anaerotignum* (*43*). Sequences belonging to the Clostridiaceae family have previously been reported from only one study in kefir (*7*).

*Coprococcus* spp. OTUs were noted in very small but similar frequencies in both grain samples (approx. 0.06-0.07%). To the best of our knowledge, bacteria belonging to this genus of strictly anaerobic cocci have never been previously associated with kefir grains. This genus includes four recognized species to date (*44*) and belongs to the order of Clostridiales (within the phylum Firmicutes). *Coprococcus* spp. are naturally present in human faeces and only rarely associated with human clinical specimens. Similarly, *Blautia* spp. OTUs were noted in very small frequencies in both grains (approx. 0.06-0.07%) and have not been previously associated with kefir. *Blautia* spp. are members of the gut microbiota and, according to recent findings, their relative abundance in the human gut is inversely associated with visceral fat accumulation in adults (*45*). *Subdoligranulum* spp. OTUs were also noted in very low frequencies in both grains (approx. 0.05%); to date, there is only one recognized species (*S. variabile*) within this genus of strictly anaerobic, Gram-negative, gut bacteria (*46*), with no previous association with kefir grains.

To our knowledge, *Acinetobacter johnsonii* (0.064% OTUs in kefir A and 0.016% in kefir B) has only been found in Malaysian kefir (*20*), although other members of the genus have been reported in Turkish (*17*) and Tibetan (*14*) kefir. Unlike other well-known pathogenic species of the genus (*A. baumannii*, a severe hospital-acquired pathogen), *A. johnsonii* is considered part of the normal human skin flora and has only rarely been associated with human disease (*47*), whereas recently, it has been shown to have the capacity to degrade polycyclic aromatic hydrocarbons (*48*).

Additional OTUs belonging to bacterial genera and species mostly associated with the gastrointestinal tract of humans and animals (*e.g. Olsenella* spp., *Allobaculum* spp*., Ruminococcus* spp., *Shuttleworthia* spp., *Phascolarctobacterium* spp., *Paraprevotella* spp. *Parabacteroides* spp., *Oscillospira* spp., *Alistipes* spp., *Flavonifactor* spp., *Roseburia* spp. *Collinsella* spp.) and never been previously associated with kefir, were also noted at rare (<0.05%) frequencies (Table 3). Whether some or all of these microorganisms are naturally present in kefir grains or came in as contaminants from human handlers during sequential kefir grain propagation (*i.e.* over the years during artisanal kefir making) remains unknown. Recent findings, however, imply that specific strains or species within some of these bacterial genera may be positively (*49*) or negatively (*50*) associated with human health. The microbiota of kefir drinks and their corresponding kefir grains can be quite different. The bacterial population of kefir milk is more consistent and less diverse than that of the corresponding kefir grains (*7*). Since we did not test kefir drinks made by the two grains, it is not known whether these minor species or genera will be present in the corresponding kefir drinks.

## CONCLUSIONS

In this study, the bacterial composition of two Greek kefir grains originating from geographically distant areas (Athens and Crete) were evaluated using a high-throughput, sequencing-based approach. The study provided for the first time an in-depth analysis of the bacterial diversity and species richness of kefir grains in Greece. In terms of bacterial populations, both kefir grains were dominated by three species of lactobacilli, with *Lb. kefiranofaciens* being the principally dominant species. However, in contrast to the small variety of dominant species, a great variety of sub-dominant genera and species were identified. Based on published scientific literature, most of this sub-dominant bacterial flora has been associated with the gastrointestinal tract of humans and animals and has never been identified as part of the kefir community worldwide. Differences between the bacterial profiles of the two grains were very small, indicating a high homogeneity despite the distant geographic origin. This study provides novel data on the bacterial ecology of Greek kefir. The detailed composition of its microbiota will be valuable in order to screen for beneficial strains from this traditional probiotic dairy product.
